# A Peak-Clustering Method for MEG Group Analysis to Minimise Artefacts Due to Smoothness

**DOI:** 10.1371/journal.pone.0045084

**Published:** 2012-09-14

**Authors:** Jessica R. Gilbert, Laura R. Shapiro, Gareth R. Barnes

**Affiliations:** 1 School of Life and Health Sciences, Aston University, Birmingham, United Kingdom; 2 The Wellcome Trust Centre for Neuroimaging, University College London, London, United Kingdom; Wake Forest School of Medicine, United States of America

## Abstract

Magnetoencephalography (MEG), a non-invasive technique for characterizing brain electrical activity, is gaining popularity as a tool for assessing group-level differences between experimental conditions. One method for assessing task-condition effects involves beamforming, where a weighted sum of field measurements is used to tune activity on a voxel-by-voxel basis. However, this method has been shown to produce inhomogeneous smoothness differences as a function of signal-to-noise across a volumetric image, which can then produce false positives at the group level. Here we describe a novel method for group-level analysis with MEG beamformer images that utilizes the peak locations within each participant’s volumetric image to assess group-level effects. We compared our peak-clustering algorithm with SnPM using simulated data. We found that our method was immune to artefactual group effects that can arise as a result of inhomogeneous smoothness differences across a volumetric image. We also used our peak-clustering algorithm on experimental data and found that regions were identified that corresponded with task-related regions identified in the literature. These findings suggest that our technique is a robust method for group-level analysis with MEG beamformer images.

## Introduction

The use of magnetoencephalography (MEG) as a research tool for brain-imaging in both normal and clinical populations is burgeoning. With advances in signal processing, beamforming has gained traction as a meaningful approach to source-localization in MEG. In beamforming, a weighted sum of field measurements is used as a spatial filter to tune an estimate of neural activity (i.e.,power) in a pre-specified time and frequency band window on a voxel-by-voxel basis. This produces a whole-brain volumetric image of signal power change which can be used for group-level analyses.

One problem in conventional MEG group analysis is that individual beamformer images are not homogeneously smooth; the images are information rich around strong sources, yet very smooth elsewhere [1], [2]. These smoothness differences have been found to range over two orders of magnitude within an image [3]. This inverse relationship between source strength and smoothness can lead to unpredictable effects at a group-imaging level. For example, at moderate signal strengths, artefactual group effects can occur. These arise because the true peaks within each source reconstruction have broad maxima (and sidelobes) whose shapes differ across participants. Through the overlap of these smooth maxima (or their sidelobes), secondary, apparently disconnected peaks can arise at a group level. A related problem of non-isotropic or inhomogeneous smoothness has been studied in the context of fMRI to correct for cluster size statistics in cases where, for example, the underlying isotropic image has been inhomogeneously resampled onto a cortical surface [4], [5]; indeed, similar solutions have been proposed for MEG [2], [6]. These solutions based on random field theory assume that voxel-to-voxel covariance can be summarized by local smoothness measures. However, the relationship between two image voxels in MEG is not just a function of their proximity (as in fMRI/PET), but also of the orientation of the dipole at that location, and therefore covariant voxels are not necessarily part of the same contiguous cluster. This is an inevitable problem in MEG source reconstruction where a large number of voxel estimates are made from a small number of channels.

In this paper, we try to step around this reconstruction problem by compressing the volumetric image to a point list of local maxima, which in turn simplifies the statistics. This is advantageous as one often ultimately wishes to interrogate individual participant beamformer estimates of electrical activity, which have been shown to be only truly reliable at the image peaks [3] (note that a similar approach has been used previously for a dipole fit analysis [7]; see discussion section for a full comparison). In brief, we assume that, under the null hypothesis, rank-ordered (e.g., by power) image peaks across participants will be no more closely grouped than any random selection of peaks.

The paper is divided into three sections. In the first section, we describe the peak-clustering algorithm and define a method for correcting for multiple comparisons when testing over a range of peaks for group-level effects. In the second section, we compare our peak-clustering algorithm against SnPM using simulated data. In the third section, we utilize our algorithm to test for group-level effects in experimental data.

## Methods and Results

### Peak Clustering Algorithm

To compare the distribution of the M top-ranked image peaks (per person) over a group of participants against any random selection of peaks, we used the following algorithm. (The matlab code is available from the corresponding author on request.):

1. Rank order the image peaks for each participant and store their corresponding locations. Since the test is based on rank order, the user must specify an interest in positive or negative peaks. The data presented in this manuscript used normalized t-tests between conditions to create images.
2. Take the coordinates of the top M peaks from each of N participants. Construct the smallest possible ellipsoid that contains a single peak from each participant. The issue here is that the top peak in participant 1 may be at the same location as the 3^rd^ peak in participant 3, etc. By selecting from M peaks, one trades off the precise peak order against spatial resolution (see later).
3. Establish if this ellipsoid is smaller (in terms of the major radius) than one would expect by chance. The computation of this radius under the null hypothesis is done by randomly assigning ranks to peak locations and repeating step 2 a large number of times (e.g., 500 in this paper). This produces a distribution of radii which one would expect due to chance (if peak rank were not important).

To give a simple example, how likely is it that the image maxima for ten participants (N = 10, one peak so M = 1) are within 1 cm of one another? To answer this, one can compute how close the image maxima will be by chance by simply taking a random image peak from each participant and repeating this process to get a null distribution of ellipsoid radii. Now one computes the same size metric using ranked peaks from each participant, then reads off the number of randomly drawn ellipsoids that are smaller than this (e.g., p<0.01).

#### Ellipsoid computation

For a given number of participants (N) and peaks (M), a k-means clustering procedure was iteratively used to derive M separate ellipsoids (ideally each of N points) from N*M points. Clusters were trimmed such that each set contained at maximum one point per participant (selecting the point closest to the centroid). At the end of the iterative procedure (typically 30 iterations), one is left with a set of the smallest (based on standard deviation of the point list) clusters for varying numbers of participants (from a user specified minimum up to a maximum of N). For these point lists, ellipsoid axes were computed from the eigenvectors and the standard deviation in each direction (and hence the 95 percentiles) computed from the corresponding eigenvalues.

### Correcting for Arbitrary Number of Peaks

The peak clustering algorithm requires some a-priori selection of the parameter M, or the number of top-ranked peaks to consider in the analysis. Typically, therefore, it is necessary to test a range of values of M, and hence there is a corresponding multiple comparisons penalty. In this section, we examine the dependence of our results on this parameter and propose an approximate heuristic for dealing with it in the future.

**Figure 1:**
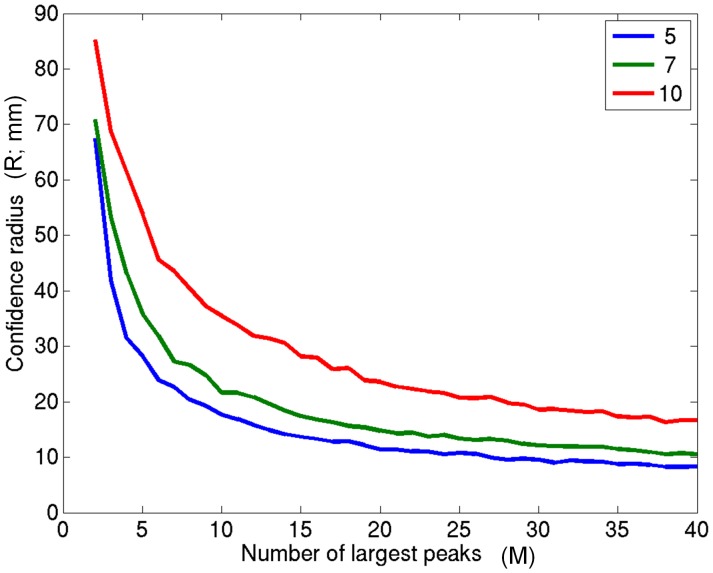
Dependence of the confidence radius on parameter M. The relationship between the number of peaks used (M) and the 95% significant (maximum) radius of the confidence ellipsoid (in mm) for subgroups of N = 5 (blue), 7 (green) and 10 (red). Intuitively, the larger the N, the larger the size of the cluster one would expect to occur by chance. In contrast, the larger the number of peaks per subject (M) considered, the easier it will be to reach a given cluster size, hence the 95% threshold decreases as more peaks are included in the analysis.

Figure 1 shows the dependence of the 95^th^ percentile of the confidence radius (*R*) (maximum radius (in mm) of the ellipsoid defining the confidence volume) on *M* for positive peaks in our experimental data analysis (see below for more information on the experimental study). Statistics are automatically produced for all subgroups from N = 5–10 participants but only N = 5, 7, and 10 are shown here for clarity. Intuitively, the smaller the number of subjects (N), the smaller an ellipsoid will be by chance (e.g., in the case of just 2 subjects, one could imagine that some peaks will be almost adjacent by chance).

**Figure 2:**
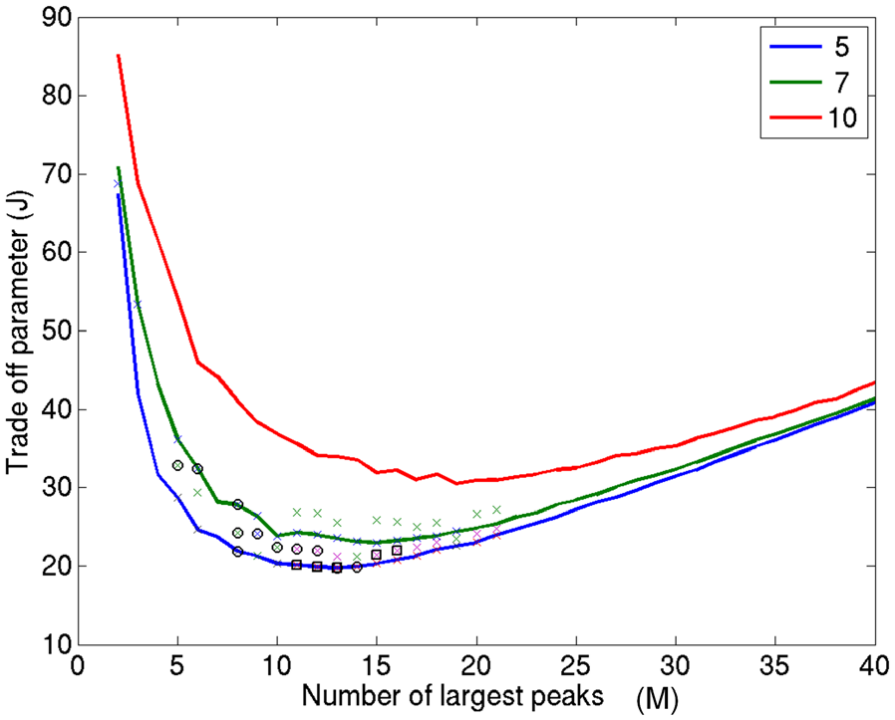
Peak amplitude and anatomical consistency trade-off. A plot of the heuristic 
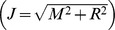
 to optimize the balance between peak magnitude and anatomical consistency across subjects. J increases for large numbers of peaks (where there is a very tight distance threshold (R) on how close the peaks must be) and also increases when M is small due to the corresponding decrease in anatomical specificity (due to increase in threshold R shown in Figure 1). Alternatively, one can choose to test a range of M (2–30 in this case), produce significant clusters (for each M; shown by crosses), and then correct for multiple comparisons. After multiple comparison correction (for M), two significant clusters were found which are denoted by the circles and squares around these points. These are the same two clusters identified in our experimental data.

The parameter *M* determines the trade-off between the importance assigned to rank order and the importance assigned to tight clustering of peaks across participants. If there is high importance assigned to rank order (smaller *M*), then relatively larger clusters of peaks across participants will be acceptable (although these may have little anatomical consistency). However, if the effect in question does not reach the top *M* peaks in most participants, it will be completely missed by the analysis. By contrast, if M is set to be too large, then the inclusion of many superfluous (i.e., low rank) peaks will mean that a very tight spatial distribution is required to distinguish a functionally meaningful cluster from one occurring by chance. This is an analogous problem to the choice of image smoothing parameters in fMRI, and analogously the choice depends on the question asked. As a starting point, we propose a simple heuristic to choose a value of *M* which balances dependence on peak rank against cluster size. If we take the knee of the curve in Figure 1 to represent some optimal balance between dependence on peak magnitude (small *M*) and anatomical consistency across participants (small *R*), we can compute a parameter J which quantifies the distance of the curves from the knee,
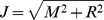
where M and R are the number of peaks and the confidence radius respectively. Now plotting *J* against *M* gives a curve with a clear minimum (see Figure 2). For each sub-group (N), crosses on the curve indicate that at least one significant (p<0.05) cluster was found for this choice of M when analyzing positive peaks. Importantly, and giving some validiation of our choice of heuristic, these significant excursions predominate around the minimum of the function.

The next problem is how to set an appropriate significance level. There is a single univariate null hypothesis–that the peaks are clustered by chance. However, as we change (increase) M, we are re-testing the same hypothesis with different subsets of data. Hence, a multiple comparisons penalty is necessary. One simple solution would be to only examine the function minima at each value of N. One problem here is that the minima are relatively flat and the smoothness depends on the number of random permutation steps performed, which is processing intensive. Also, one can see from Figure 2 that each subgroup curve N has a different optimal M value (the larger the number of participants in the group, the larger the optimal number of peaks).

Another possibility is to consider the range of M which defines this minimum. This approach does not rely on the identification of minima (so it is more robust) and can be computed for all N at once. However, there is a multiple comparisons penalty. It is important to note, however, that a completely new (i.e., independent) set of data is only introduced each time the number of peaks is doubled.

Making a Bonferroni correction, the significance level should be decreased by a factor each time the number of peaks is doubled. This means that the test wise error rate to give a family wise error rate of 0.05 is based on the following Bonferroni correction:
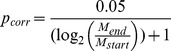
where log_2_ is log to the base 2, p_corr_ is the corrected significance level and M_start_ and M_end_ define the range of M we pre-specify an interest in. The circles and squares around the crosses in Figure 2 show the two significant ellipsoids found after multiple comparisons correction for the range of peaks tested (for M_start_ = 2 and M_end_ = 30).

### Measuring Algorithm Performance: Simulated Data

In order to test algorithm performance against some ground truth we simulated a single dipolar source across a group of participants. The same single sphere head model and sensor locations were used for each simulated participant. System white noise was simulated at 10 fT/sqrt (Hz) over a bandwidth of 80 Hz. Data for 10 participants were simulated, differing only in the simulated source location and white noise realization. In each simulated participant, a random seed location was generated, drawn from a Gaussian distribution of standard deviation 5 mm, centered on MNI location x = 52, y = −29, z = 13. The nearest canonical mesh location [8] to this seedpoint and the corresponding surface normal were used to set the location and orientation of the single simulated dipole in each participant. Our simulated sources were normal to the cortical mesh, but as location was jittered, both source location and orientation changed over participants. The dipolar source was driven with a 40 Hz sinusoid over a period of 200 ms (sample rate = 200 Hz). The source was active for 30 of 60 epochs and a linearly constrained minimum variance (LCMV) beamformer was used to produce a volumetric beamformer image of the change in power in the 0–300 ms, 0–80 Hz band in terms of a normalized difference (or pseudo-t) image [9] on a 10 mm grid. The beamformer has been described extensively [2], [3], [9], [10], and an abbreviated version is presented here.

The beamformer is simply a spatially filtered expression of the MEG sensor data.
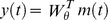
where *W* is a vector of weighting coefficients and m(*t*) is the measurement vector at time *t*. To obtain the weighting coefficients, power is minimized over the covariance window subject to the constraint of unit gain at a specified coordinate θ:



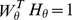
where *H* is the forward solution for an equivalent current dipole (ECD) at coordinates and orientations specified by the vector θ. The solution to the equation is:



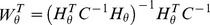
where *C* is the covariance matrix of the measurements calculated over the specified covariance window (T_cov_). The 2 (i.e., single-sphere) or 3 (i.e., multiple spheres) orthogonally oriented components of W at each location can be estimated independently to produce a vector beamformer. In this case, we used a scalar beamformer in which optimal source orientation at each voxel was estimated through the method of Sekihara et al. [11]. A normalized source power estimate can be obtained over any test period (within the covariance window) through the estimation of the sensor level covariance matrix C_test_ over this period, and an estimate of the sensor noise ε_test_ (in this case, we used identity) matrix over this period:



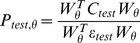



We should note that in the experimental data analysis stage, we used the proprietary software (SAM) to analyze the data [9]. This computes separate covariances (and hence weights) for both active and passive periods. In the simulation stage, however, we computed a single covariance matrix (based on both active and passive periods), but as there was only white noise in the passive period, this should have marginal effect on the power difference calculation (see discussion).

Different participant groups were constructed by drawing 8 of these 10 images randomly twenty times. For each participant group, we used SnPM (multiple participant, one sample t-test, variance smoothing 25 mm) to identify significant (family wise error = 0.05) positive effects across the normalized power difference images. Using the peak clustering algorithm, we used the same data to look for clusters within the top 5 image peaks that were smaller than one would expect by chance (i.e., M = 5 peaks, N = 8 participants). For each simulated group, we compiled a list of the significant local maxima (p<0.05 corrected) in the SnPM images and a list of the centers of the peak-clusters deemed significant. We classed a hit as a peak/ellipse center closer than 20 mm to the initial MNI seed location and a miss to be any significant peak or ellipsoid center outside this range. The peaks were defined by local image maxima identified using the SPM function spm_max based on 18 neighbors. This means that two local maxima can be as close as a single (non-maximal) voxel apart.

### Measuring Algorithm Performance: Experimental Data

**Figure 3:**
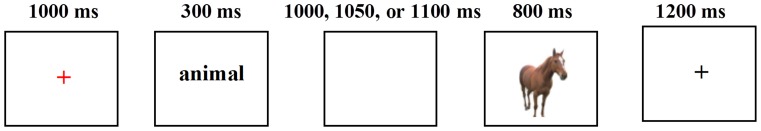
Example experimental data trial. During study 1, participants were shown a 1000 ms red fixation cross, followed by a 300 ms category probe. After a variable (1000, 1050, or 1100 ms) delay interval, participants were shown a target object for 800 ms.

We assessed the performance of our peak-clustering algorithm on experimental data. In our experiment, ten right-handed volunteers (Mean Age = 29.4 years, range = 20–36 years; 2 males) gave written informed consent following Aston University ethical guidelines and participated in the MEG study. The protocol was approved by the Aston University Institutional Review Board and complied with all guidelines expressed in the Declaration of Helsinki. Briefly, participants (N = 10) performed a superordinate-level categorization task on pictures of objects drawn from 3 living and 3 nonliving categories (see Figure 3). A total of 78 pictures were selected, half of which depicted a living object and half a nonliving object. Each picture was shown twice, half with a congruent label and half with an incongruent label. Therefore, a total of 156 trials were shown during the scan. The order of trial presentation was randomized across participants. We recorded neuromagnetic data at a 600 Hz sampling rate with a bandwidth of 0–150 Hz using a CTF 275 MEG system (VSM MedTech Ltd., Canada) composed of a whole-head array of 275 radial 1st order gradiometer channels housed in a magnetically shielded room (Vacuumschmelze, Germany). Synthetic 3rd gradient balancing was used to remove background noise on-line. Fiducial coils were placed on the nasion, left preauricular, and right preauricular sites of each participant. These coils were energized before each run to localize the participant’s head with respect to the MEG sensors. Total head displacement was measured after each run and could not exceed 5 mm for inclusion in the source analyses. Prior to scanning, participants’ head shapes and the location of fiducial coils were digitized using a Polhemus Isotrak 3D digitizer (Kaiser Aerospace Inc.). These were then coregistered to high-resolution T1-weighted anatomical images for each participant acquired with a 3-Tesla whole-body scanner (3T Trio, Siemens Medical Systems) using in-house coregistration software.

Data for each participant were edited and filtered to remove environmental and physiological artefacts. A LCMV beamformer was then used to produce 3-dimensional images of cortical power changes [9]. We utilized a wide frequency band (1–80 Hz) to compute source power from 120–220 ms after stimulus onset (i.e., a 100 ms window surrounding the M170), directly contrasting living (‘active’) to nonliving (‘control’) target objects. Spectral power changes between the ‘active’ and ‘control’ periods were calculated as a pseudo t-statistic [9]. Each participant’s data were then normalized and converted to Talairach space using statistical parametric mapping (SPM99, Wellcome Department of Imaging Neuroscience, London, UK, http://www.fil.ion.ucl.ac.uk/spm) for group-level comparisons.

We used SnPM (multiple participant, one sample t-test, variance smoothing 6, 12, and 24 mm) to identify significant (family wise error = 0.05) positive effects across the normalized power difference images. We also used our peak-clustering algorithm to test over a range of M values from M = 2 through 40 (we utilized only positive peaks in the analysis), which means that in order to maintain a family wise error rate of 0.05, our test wise error rate was adjusted to p = 0.0094. After multiple comparisons correction, we were left with a number of significant clusters of peaks (see Table 1). The remaining volumes decreased in size spatially as M increased so if the same region was identified as showing a significant difference across a range of M values, we selected the region for reporting purposes that yielded the largest N. In some cases, several M values yielded the same N. We then chose the volume for reporting purposes that had the smallest spatial extent (in terms of the major radius).

**Table 1:** Experimental Results.

Location	BA	N	M	Coordinates ofCenter (x, y, z)	Volume(mm^3^)	Major Radius(mm)	Mean Value	p-value
Left Inferior Occipital Gyrus	19							
		6	5	−37, −83, −10	7,777	22.8	1.95	0.00
		7	6	−35, −85, −14	16,488	28.4	1.91	0.01
		5	8	−49, −73, −9	458	17.4	1.71	0.01
		6	8	−34, −85, −9	2,352	15.7	1.99	0.00
		**7**	**8**	**−40, −81, −7**	**4,891**	**22.3**	**1.84**	**0.01**
		6	9	−34, −85, −9	2,352	15.7	1.99	0.00
		6	10	−34, −85, −9	2,352	15.7	1.99	0.01
		6	11	−34, −85, −9	2,352	15.7	1.99	0.00
		6	12	−34, −85, −9	2,352	15.7	1.99	0.01
		5	13	−35, −85, −6	1,227	11.6	1.99	0.00
		5	14	−35, −85, −6	1,227	11.6	1.99	0.01
Right Superior Temporal Gyrus	38							
		5	11	49, 3, −14	406	12.6	1.78	0.00
		5	12	49, 3, −14	406	12.6	1.78	0.01
		5	13	49, 3, −14	406	12.6	1.78	0.01
		**6**	**15**	**49, 5, −14**	**1,839**	**12.4**	**1.70**	**0.01**
		6	16	49, 5, −14	1,839	12.4	1.70	0.01

The two regions identified by our peak-clustering algorithm as showing a significant group-level difference between living (active) and nonliving (control) objects from 120–220 ms using a wide frequency band (1–80 Hz) (Note: the analysis included only positive peaks). Here, we show the 11 ellipsoids centered in left inferior occipital gyrus and the 5 ellipsoids centered in right superior temporal gyrus (arranged by increasing M values). Note that the highlighted ellipsoids (bold) are the regions used for reporting purposes. BA = Brodmann area. N = number of participants (out of 10) having a peak within the volume. M = number of peaks used to identify the region; p = corrected p-value.

### Simulation Results

**Figure 4:**
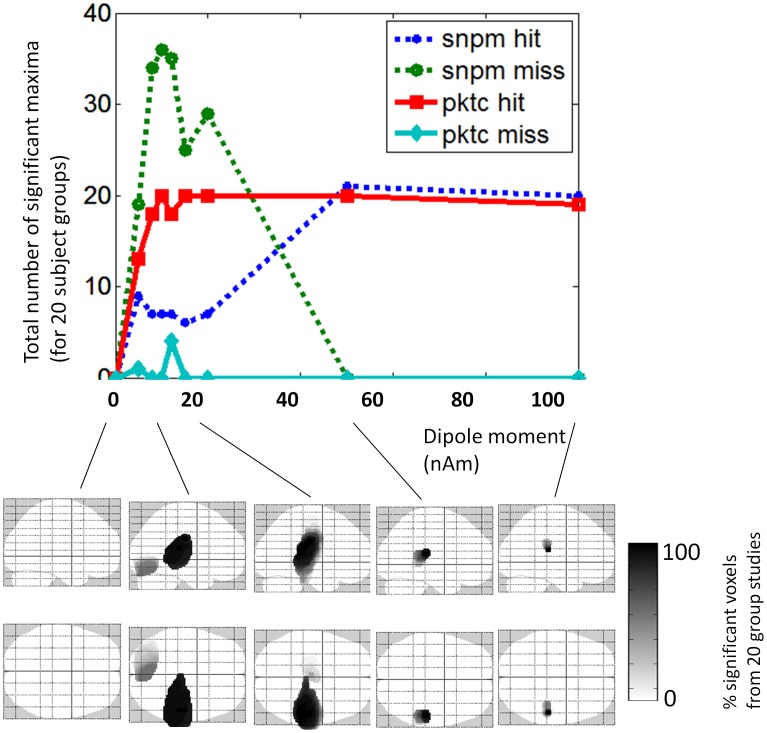
Data simulation findings. Top panel shows the total number of significant local maxima over 20 simulated subject groups (with a single simulated source) identified using SnPM (dotted) and the peak clustering method (solid) as source magnitude is increased. Local maxima within 2 cm of the simulated source are defined as hits and those greater than 2 cm misses. Note that both methods consistently identify the correct source location at high SNR (20 hits, 0 misses) but that SnPM tends to produce a large number of artefactual significant regions at moderate SNR. This error rate is due to the smoothness of the beamformer images that gives rise to statistically significant overlapping side-lobes. These effects are shown in the lower panel, where maps of the percentage of significant voxels (from the 20 groups) are shown in the glass-brain.

Figure 4 (top) shows the number of hits and misses summed over the 20 participant groups for the two methods. At moderate SNR, the number of misses for SnPM is much higher than for the peak-clustering approach. This is due to extra peaks appearing in the SnPM images due to artefacts of smoothness. Figure 4 (bottom) shows binarized (thresholded at p<0.05 corrected) SnPM significance images summed over the 20 groups (and then normalized to the maximum count). That is, the maps show the spatial distribution of significant regions and the grey scale shows their relative frequency (over groups). For moderate source strengths (i.e., 10–20 nAm), one can see the appearance of extra significant clusters, which give rise to the inflated miss rate. Note that these misses are not false positives in the statistical sense, but simply image features that persist over participants due to the source reconstruction method. The peak-clustering approach is immune to these extra features as there are no consistent local maxima in these vicinities across participants. In this particular example, the peak-clustering approach is also more sensitive (i.e., a maximum of 20 hits reached before SnPM). Note, however, that in this case we have prior knowledge of how many of the top peaks to consider.

### Experimental Results

**Figure 5:**
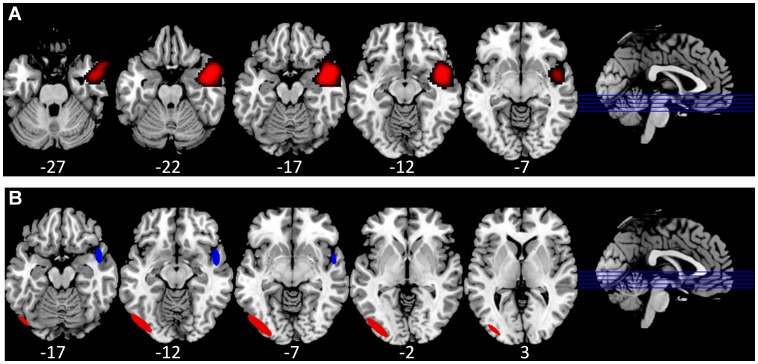
Experimental data findings. A) The region in right anterior middle to superior temporal gyrus identified by the SnPM analysis as showing significantly greater power for living compared with nonliving objects. B) The two regions identified by the peak-clustering algorithm as showing significantly greater power for living compared with nonliving objects. Red = Inferior Occipital Gyrus; Blue = Superior Temporal Gyrus. The sagittal images show the approximate slice locations (z coordinates are given below each slice) shown on the corresponding axial image (at right, blue lines, arranged inferior to superior) on a template brain.

The SnPM analysis did not identify any regions showing significant positive power differences when using 6 or 12 mm variance smoothing. However, a single region centered in right anterior middle to superior temporal gyrus (Talairach coordinates of center = 48, 3, −18) was identified when we set variance smoothing to 24 mm (see Figure 5). The peak-clustering analysis of positive peaks identified two separate regions showing greater power for living objects (see Figure 5). The region with the largest N was centered in left inferior occipital gyrus, and using the top 8 positive peaks in each image, 7 of our 10 participants were found to have a peak falling within the region (major radius = 22.3 mm, mean value = 1.84). In addition to this region, when using the top 15 positive peaks in each image (i.e., a less stringent magnitude criterion), 6 of our 10 participants were found to have a peak falling within a region in right anterior superior temporal gyrus (major radius = 12.4 mm, mean value = 1.7). This region overlapped with the region identified in the SnPM analysis.

## Discussion

We have presented a peak-clustering algorithm for group-level analysis with MEG beamformer images. Our algorithm determines whether a range of image peaks (M) is closer than expected by chance. We compared the peak-clustering algorithm performance to a more traditional group imaging method (SnPM) and found the algorithm to be robust to artefacts of smoothness that can give rise to erroneous MEG beamformer group effects. There is an important distinction here between false positives due to type 1 error and the effects we are trying to correct for. Both SnPM and the peak-clustering algorithm have, by definition, the correct type 1 error rate (as it is set in both cases by permutation). Neither is there a problem with SnPM. The issue we are trying to correct for here is one of source reconstruction, where a small number of data channels are projected into a large number of voxels, resulting in images which are very smooth in certain regions. It is therefore a way of pruning away redundant information from beamformer images to reduce the likelihood that these smooth and information sparse regions of source space contribute to the group effect.

Our approach is similar to a dipole fit analysis approach used previously [7]. In the Litvak paper, the focus was on identifying the differences between experimental conditions through the permutation of condition labels to create sensor-time and dipole fit clusters. By comparing this null (e.g., in terms of distances between dipole clusters) to the true distribution, the authors were able to put a significance level on how likely the conditions were to be the same. The main differences between the Litvak technique and our own are that we shuffle peak rank rather than data labels, and we do not have a theoretical source model (e.g., 1 or 2 dipoles) but are looking for consistency over images which may contain large numbers of sources. That said, the same approach of shuffling data labels (rather than peak rank) to generate the null could also be used here to make inferences on whether the ellipsoids due to separate stimulus conditions were any larger than that due to their mixture.

As mentioned previously, in the algorithm we are effectively trying to compensate for the few (i.e., channel) to many (i.e., voxel) mapping in M/EEG volumetric source reconstruction. This problem is exacerbated in beamformer analyses because of the dependence of spatial resolution not only on system sensitivity, but also on source power [1], [2]. An additional problem not addressed here is that in the SAM implementation used for the experimental data (i.e., CTF version), different covariance matrices are used to construct different beamformer weights for different task labels (in contrast to a single set of weights for all tasks, cf. [2]). That is, the statistical image is a test between two non-stationary images. For the purposes of this study, the distinction is not important because either way the images are inhomogeneous. We are not proposing a new or improved inversion algorithm, simply a method by which some of the smoothness inhomogeneities (due to any volumetric reconstruction) can be discarded. Also, for our beamformer analysis, we used no regularization. This gives maximum spatial resolution at the expense of noisy images and time-series estimates. It would also give rise to the maximum number of peaks per image. A higher regularization constant would reduce the number of peaks, removing some that were potentially just due to sensor noise, but potentially risk discarding signal peaks. At some ideal level, one would expect the highest ratio of signal to noise peaks [12]. We do know that there can be a maximum N channels minus 1 nulls in the beamformer image [10]; so, for a simple (i.e., unregularized) power image one would expect approximately the same number of local maxima.

The algorithm requires a parameter that defines the number of top-ranked peaks to consider (M) for each participant. This parameter has important implications for cluster size. Since the algorithm first computes chance volume sizes using a random selection of peaks, using a small number of peaks can produce a large cluster size for the null distribution. Rather than arbitrarily determining the number of peaks for the algorithm to consider, we developed a heuristic that balances peak rank against cluster size that requires the user to test over a range of M values and use a Bonferroni correction for multiple comparisons. For example, to maintain a family wise error rate of 0.05 when testing over 38 P-values (i.e., 2–40), the test wise error rate becomes 0.0094. It is important to note that the choice of M can be made based on simulations or on the data themselves, as long as an appropriate multiple comparisons correction is made. For this reason we had expected the algorithm to be more conservative than volumetric approaches (like SnPM), but by only dealing with the image in its compressed point-list form, rather than all voxels, we have also considerably reduced the multiple comparison correction necessary. This may explain why, counter to our expectation, the algorithm picked out significant features in the experimental dataset that were not apparent in (the volume corrected) SnPM tests.

In our experimental study, participants were required to perform a superordinate-level categorization task on pictures of living and nonliving objects. The SnPM analysis yielded mixed results based on the variance smoothing used. When using both 6 and 12 mm, no regions survived statistical significance. However, when using 24 mm, a single region in right anterior middle to superior temporal gyrus showed significantly greater power for living than nonliving objects. Using the peak-clustering algorithm, we also found a significant cluster of activity in right anterior superior temporal gyrus, overlapping with the region identified by the SnPM analysis. In addition, we identified a region in left inferior temporal gyrus showing greater power for living than nonliving objects, which we did not find in our SnPM analysis. In order to determine whether the SnPM analysis yielded a peak in left inferior temporal gyrus that simply did not survive whole-brain correction, we looked at the t map produced in our SnPM analysis. We found a cluster of activity centered in left inferior temporal gyrus (peak value = 2.95), which suggests that left inferior temporal gyrus would be significant if we performed a region-of-interest analysis (rather than a whole-brain analysis) using roughly 7 independent voxels (or ROIs). This would be in accord with our explanation that the peak clustering analysis has a less stringent multiple comparisons penalty, as it considers only a limited number of image peaks per subject (indeed for these analyses there were 8 peaks per participant). Both of these regions we would expect to be active based on previous neuroimaging studies which have suggested that the inferior temporal/occipital gyri are important for form recognition, and that reliance on visual form is more important for living than nonliving objects [13], [14]. In addition, studies have also suggested that the anterior superior temporal gyri are important for object recognition, including making fine-grained distinctions amongst objects [15]. Several studies have also suggested that identifying living objects requires greater fine-grained discrimination than nonliving objects, perhaps due to greater structural (and semantic) similarity among living than nonliving things [16], [17].

As with many non-parametric techniques, the peak clustering method sacrifices some sensitivity for an increase in robustness, and requires that some feature of interest (here, each peak) is identifiable in the majority of individuals. This would not be the case in standard random or fixed effects models in which sub-threshold effects in the individual can be picked up in the group. Allowing the algorithm to identify smaller subgroups is a matter for debate. In some cases, the objective identification of subgroups might be a useful feature of the algorithm. Forcing the algorithm to be selective to only those regions in every participant that have a local maximum makes it extremely conservative. Once could also argue that a group effect is meaningless if one does not include the whole group. Yet, in classical volumetric approaches, random effects analysis allows some heterogeneity in the effects over the population. As long as the values of N (e.g., N = 9 for a group of 10) are reported then the reader can make his/her own inference on the strength of the finding (e.g., an effect in 90% of the participants). Also, the technique will not be sensitive to truly spatially extended regions of electrical activity that are not artefacts of smoothness, as only the peaks within each image are considered in the analysis.

In sum, we have found that our peak-clustering technique offers a number of advantages over current group-level analysis approaches with MEG. The method is immune to inhomogeneous smoothness introduced by imperfect volumetric M/EEG source reconstruction and exacerbated in beamformer implementations, and indeed it makes no assumptions about the underlying image properties. In addition, the null distributions of source locations are constructed from the data itself and the randomization testing takes into account the multiple comparisons problem (for a given M). As the test is based on rank, it should be relatively robust to physiological artefacts and as a default we would leave the artefact identification until the post-hoc analyses. For example, eyeball artefacts should result in significant clusters in the eyes. Subgroup statistics are also available, so, for example, bounds for any 5 of N participants having significantly clustered peaks can automatically be tested. Finally, by providing confidence intervals on peak location, the technique would be well suited to situations in which one would like to make some spatial inference concerning peak location. For example, whether peaks from a particular subject group derive from a specific cortical location.
